# A rare case of lobular breast cancer metastasizing to large bowel

**DOI:** 10.1002/ccr3.4081

**Published:** 2021-05-04

**Authors:** Waka Yanagisawa, Sandra Krishnan, Adrian Fernandez

**Affiliations:** ^1^ Department of General Surgery Prince of Wales Hospital Sydney NSW Australia; ^2^ South East Regional Hospital Bega NSW Australia

**Keywords:** breast cancer, invasive lobular breast cancer, metastatic bowel obstruction

## Abstract

Lobular breast cancer metastasis to bowel is rare, however, when it occurs, the prognosis is poor. Possible benefits of investigation with screening endoscopy for gastrointestinal metastases are discussed in order to optimize prognosis for patients.

## Graphical abstract

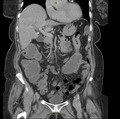

## 1 INTRODUCTION

Lobular breast cancer is rarely known to metastasize to the gastrointestinal tract.^1^ A 78‐year‐old with Invasive Lobular carcinoma in remission had partial intestinal obstruction that was discovered from metastasis to the colon. Investigation of gastrointestinal symptoms in patients managed for invasive lobular carcinoma should include possibility of gastrointestinal metastasis.

Breast cancer is the most common malignancy in women, most frequent in the fourth and fifth decades of life and comprises 27% of all cancers.^2^ Since the introduction of the national breast screening program such as in the United States, and earlier detection, only 7% of breast malignancies have metastasized at the time of presentation.^2^ According to Miller et al (2013), breast cancer most commonly metastasize to lungs, bones, liver, and brain,^2^ and in some occasions, invasive lobular carcinoma (ILC) has been demonstrated to metastasize to the gastrointestinal tract.^2^ We describe a case of a patient in remission from lobular breast cancer who presented with metastatic lobular breast carcinoma requiring an extended right hemicolectomy. As there is still a lot to be learnt in regard to metastatic spread of the disease, this case report raises the discussion regarding possible benefits of investigating patients for potential gastrointestinal metastases.

## 2 CASE REPORT

A 78‐year‐old female presented with a 1‐month history of colicky abdominal pain, abdominal distension, vomiting, and unintentional weight loss. Her past medical history included ischemic heart disease on clopidogrel and previous history of deep vein thrombosis. She recently returned a positive stool occult blood test, awaiting colonoscopy. Her past surgical history included a hysterectomy, cholecystectomy, appendectomy and notably, a right lobular Stage IIB, Grade 2 lobular breast cancer. She had a wide local excision and sentinel lymph node excision with 1/3 biopsied lymph nodes positive. Despite her positive lymph node, she was deemed lower risk lobular type, and her treatment included adjuvant radiotherapy for 6 weeks and hormonal therapy letrozole, which was discontinued after 7 months due to arthralgia severely impacting her quality of life. Tamoxifen was deemed unsuitable due to associated risk of thromboembolism and her previous history of deep vein thrombosis. She continued regular outpatient surveillance and was in remission, confirmed by a normal breast ultrasound and mammogram a year prior to presentation.

**FIGURE 1:**
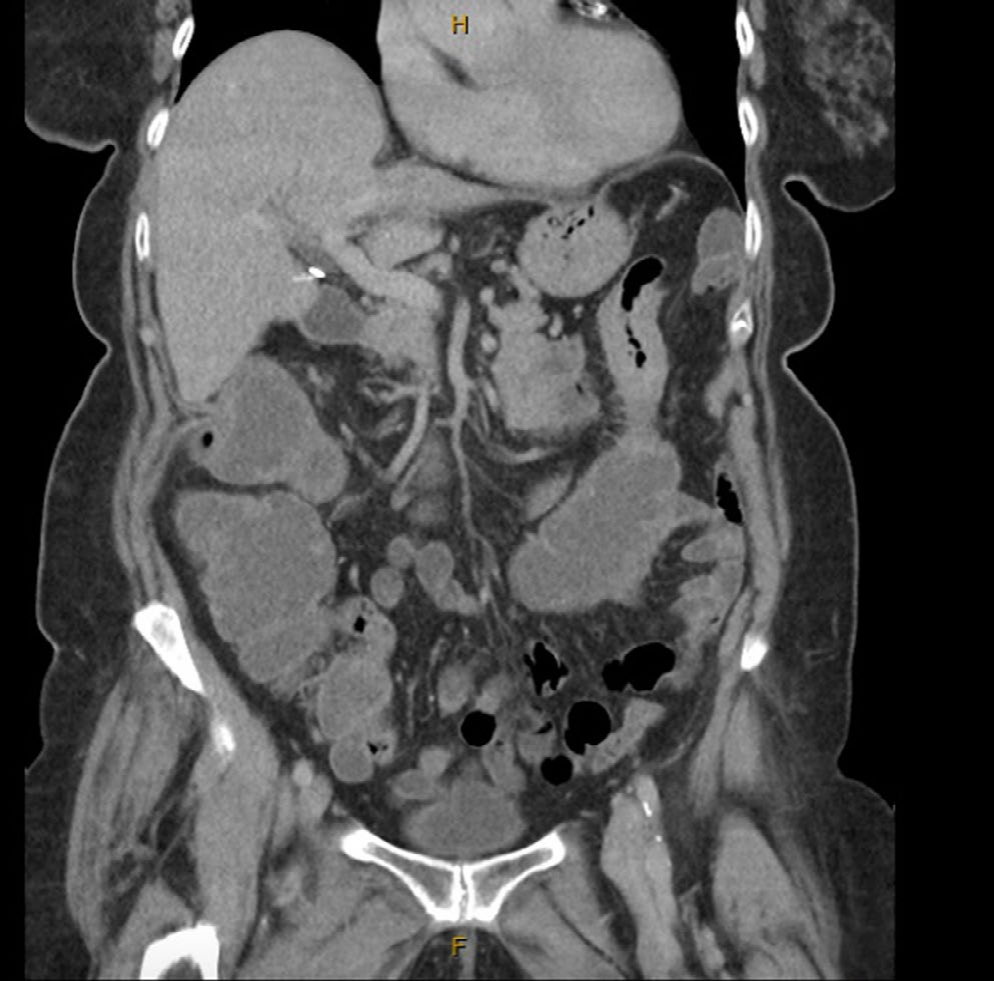
CT abdomen/pelvis coronal and sagittal view, respectively—long segment of concentric wall thickening and narrowing of the distal transverse colon with dilatation of proximal transverse colon and ascending colon

**FIGURE 2:**
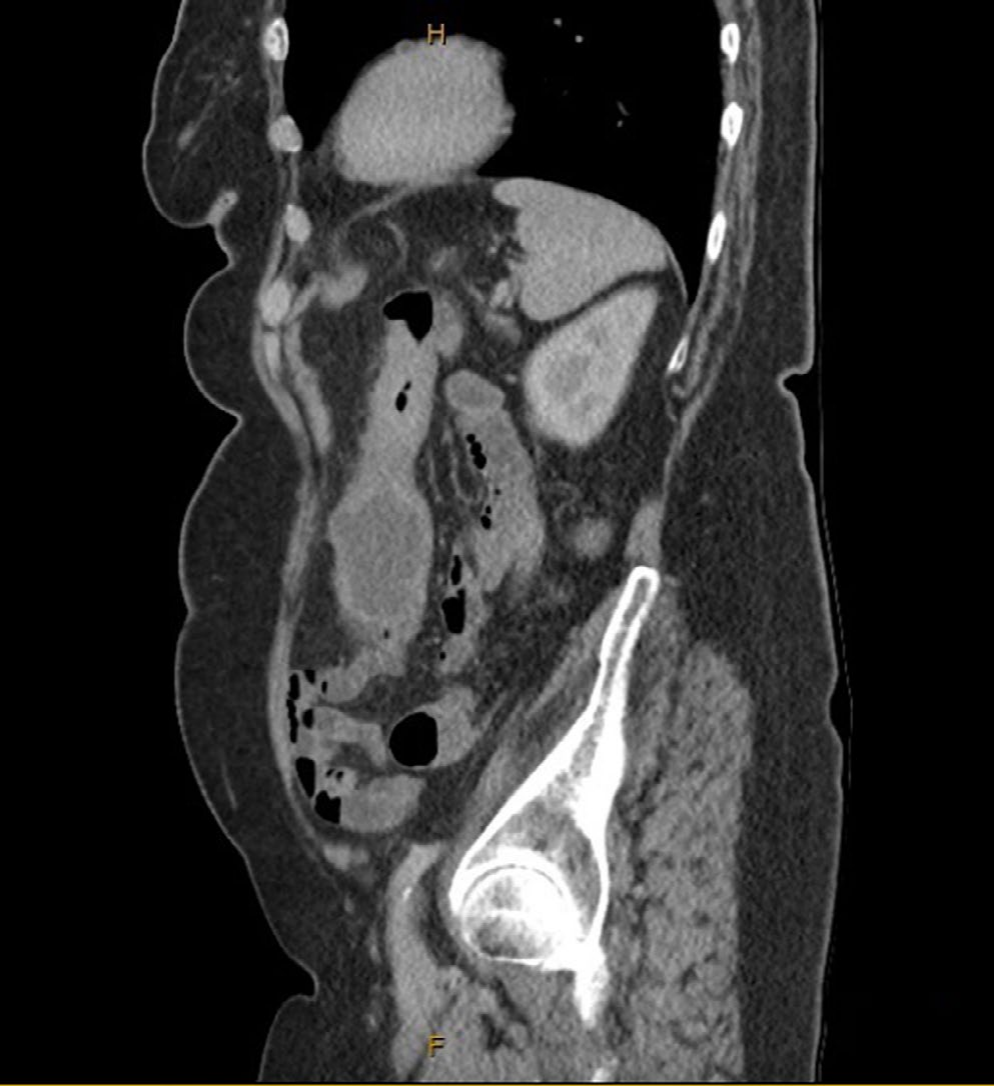
CT abdomen/pelvis coronal and sagittal view, respectively—long segment of concentric wall thickening and narrowing of the distal transverse colon with dilatation of proximal transverse colon and ascending colon

An Abdominal Computed Tomography (CT) in the Emergency Department at the time of presentation demonstrated concentric wall thickening and narrowing of the distal transverse colon with dilatation of the proximal transverse colon and ascending colon, concerning for a neoplastic lesion (Figures 1 and 2). Her CEA level was elevated at 5.8. Subsequent investigation with a colonoscopy revealed a circumferential lesion in the proximal descending colon, not typical in appearance for an adenocarcinoma. It was difficult to negotiate past the tumor and biopsies were taken. The histopathology demonstrated features of sessile serrated adenoma with some reactive changes, however, no evidence of dysplasia or malignancy. A semi‐urgent laparotomy with curative intent was performed in view of her progressive abdominal pain and distension and found the offending, thickened segment to involve the distal transverse colon and splenic flexure. An extended right hemicolectomy was performed, and her surgery was routine. The histopathology comprised of a circumferential, firm, ill‐defined partially ulcerated lesion in the transverse colon measuring 25 × 20 mm and measuring 370 mm to the proximal margin, 110 mm to the distal margin. The lesion appeared to invade through the muscularis propria into the pericolic fat by 5 mm and measures 1 mm to the closest serosal surface. Multiple lymph nodes were identified in the mesentery ranging from 2‐20 mm in maximal diameter. This was demonstrated to be a metastatic, grade 2 lobular carcinoma deposit. Lymphovascular invasion was present and three separate extranodal deposits of metastatic lobular carcinoma were seen in the mesenteric tissue. The immunohistochemistry demonstrated ER and PR positive status, HER2 FISH negative and a Ki‐67 of 18% in keeping with the previous lobular breast carcinoma histopathology.

She recovered well postoperatively, requiring a short period of rehabilitation. She was recommenced on letrozole to reduce the risk of further recurrence and associated issues including ascites and pleural effusion. She was not a candidate for chemotherapy given her frailty. A CT pan scan and bone scan did not demonstrate any other evidence of metastatic disease. Prior to further follow‐up and a planned PET scan in a tertiary center. Unfortunately, prior to further follow‐up, she passed away, 2 months postoperatively with increasing anorexia and fatigue.

## 3 DISCUSSION

**FIGURE 3:**
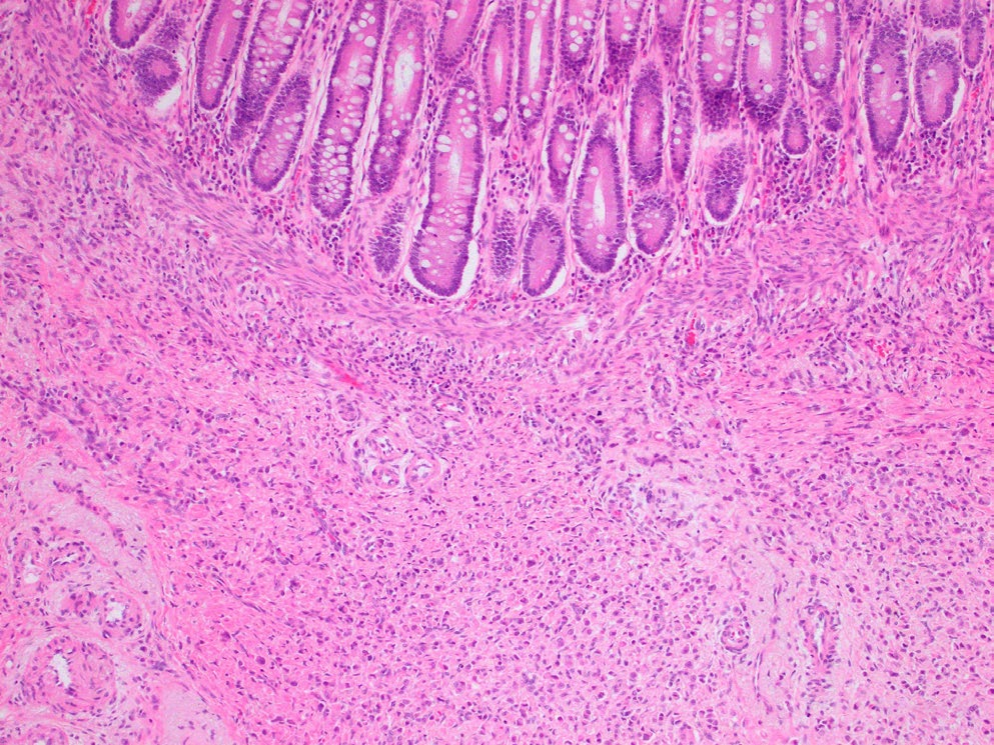
Hematoxylin and eosin stain: Hematoxylin and eosin stained section from the large bowel tumor ×100 magnification. The carcinoma cells are infiltrating upwards toward the bowel mucosa (top). The carcinoma cells are relatively small and are disposed as infiltrating cords and single cells. There is no tubule formation in this tumor. This is typical of classical lobular carcinoma of the breast

This is an interesting case of metastases to the gastrointestinal tract being the first manifestation of breast cancer metastases years following diagnosis of the primary tumor. The clinical presentation may be a specific such as abdominal pain as was a symptom in this case; however, others may present with bleeding or diarrhea as discussed in other case reports of gastrointestinal metastases of breast cancer in available literature.^3^ CT scan in this setting therefore is a useful diagnostic tool, which has been demonstrated to be a useful noninvasive assessment for assessing mural penetration.^3^ However, for definitive diagnosis, endoscopy and biopsy are required to guide management. Surgery is also often required for treatment of subacute bowel obstruction or bleeding ^3^ such as a right hemicolectomy in this case. ILC accounts for 10% of all breast cancers and is the second most common breast carcinoma.^1^, ^4^ The incidence is increasing in postmenopausal women, possibly secondary to the use of hormone replacement therapy in this age group.^4^ Breast cancer frequently metastasizes to skeletal and pulmonary systems, however, metastasis to the gastrointestinal tract seems to be more frequent in lobular histology.^5^ In a study of 2605 cases of breast cancer with metastases; 359 lobular and 2246 with ductal carcinoma, 4.5% in ILC metastasized to the gastrointestinal tract compared with 0.2% in the ductal carcinoma group.^6^ Gastrointestinal tract involvement with metastatic ILC is thought to most commonly involve stomach and small bowel, then followed by colon and rectum.^4^ According to available literature, 60% metastasizes to the stomach, 12% to the esophagus, 11% to the colon, 8% to the small intestine, and 7% to the rectum.^3^ The reason for this mechanism of spread is unclear, however, some possible reasons may include the histopathology of ILC. ILC is characterized by small cells that infiltrate the breast stroma in a single‐file “Indian‐file” pattern, which does not destroy anatomical structure nor result in a reactive connective tissue response (Figure 3), and can therefore fail to distinctly form a mass in the breast which can be difficult to identify early.^4^ Patients are characterized by poor prognostic profile and few survive longer than 2 years following metastasis.^1^, ^7^ This may account for the higher distant metastatic rate in ILCs.^8^ Other reasons for metastasis of lobular breast cancer may be explained by E‐cadherin which is crucial in the maintenance of epithelial cell polarization. E‐cadherin is a calcium‐dependent epithelial cell adhesion molecule when mutated or lost can be associated with metastases as it acts as an invasion suppressor.^9^ Deficiency of this molecule is responsible for cancer metastasis due to loss of cell to cell adhesion, with increased cell motility causing spread in blood and lymphatics.^10^ In lobular breast cancer, E‐cadherin loss has been observed in most cases but not observed in invasive ductal cancers. In some studies, E‐cadherin has been used to be reliably used as a marker to differentiate between IDC and ILC.^11^ This may account for its aggressive behavior compared with IDC.^9^ Poor prognostic factors include age over 70 years, and degree of nodal involvement, which are associated with significant increased risk of relapse and death.^12^ She had two of these factors including age and involvement of nodes which may have increased risk of poorer outcome. The uncertainty of the nature of ILC makes this an intriguing field, requiring further research and thoughtful consideration in order to most appropriately manage patients. Patients diagnosed with breast ILC and particularly those above 70 years of age should be carefully monitored for any evidence of potential gastrointestinal metastases, and if masses are identified to obtain tissue diagnosis for appropriate treatment.

## 4 CONCLUSION

Patients with gastrointestinal symptoms with known invasive lobular breast cancer should be adequately investigated for potential metastases and if any masses are identified, the importance of obtaining tissue diagnosis to rule out metastases or a second primary. Particular attention to the subtype of ILC and prognostic factors including age and nodal involvement should alert clinicians to the likely possibility for gastrointestinal metastases. Knowledge of this unusual pattern of disease will help plan appropriate treatment, in order to optimize prognosis.

## CONFLICT OF INTEREST

None declared.

## AUTHOR CONTRIBUTION

WY, SK, and AF: were all involved in reviewing the literature, prepared and edited the manuscript and all authors approved the final version of the manuscript.

## ETHICAL APPROVAL

This study does not require any ethical committee approval.

## Data Availability

Data sharing is not applicable to this article as no new data were created or analyzed in this study.
